# To-and-fro coronary flow and simultaneous dual coronary artery occlusion following Fontan surgery in pulmonary atresia with intact ventricular septum

**DOI:** 10.1093/ehjcr/ytaf208

**Published:** 2025-04-25

**Authors:** Kodai Yamazaki, Kenji Kada, Kazumasa Suga, Tomoyuki Ota

**Affiliations:** Department of Cardiology, Japan Community Healthcare Organization Chukyo Hospital, 1-1-10 Sanjyo, Minami, Nagoya, Aichi 457-8510, Japan; Department of Cardiology, Japan Community Healthcare Organization Chukyo Hospital, 1-1-10 Sanjyo, Minami, Nagoya, Aichi 457-8510, Japan; Department of Cardiology, Japan Community Healthcare Organization Chukyo Hospital, 1-1-10 Sanjyo, Minami, Nagoya, Aichi 457-8510, Japan; Department of Cardiology, Japan Community Healthcare Organization Chukyo Hospital, 1-1-10 Sanjyo, Minami, Nagoya, Aichi 457-8510, Japan

## Case description

**Figure 1:**
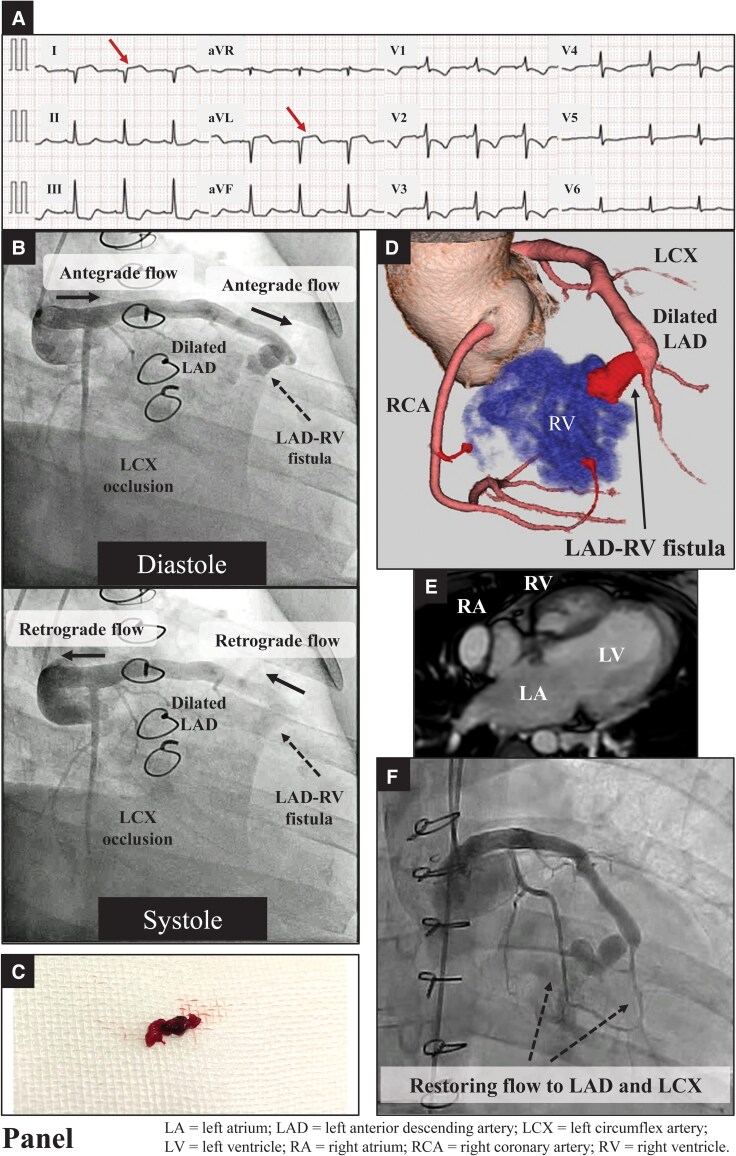
(*A*) Electrocardiography showing ST-segment elevation in leads I and aVL (arrows). (*B*) Coronary angiography showing a to-and-fro flow pattern in the dilated left anterior descending artery during both the diastole and systole phases, with antegrade flow during diastole and retrograde flow during systole. Left circumflex artery occlusion is also visible. (*C*) Retrieved red thrombus from the thrombectomy procedure. (*D*) Three-dimensional computed tomography reconstruction demonstrating the anatomical relationship between the dilated left anterior descending artery, left circumflex artery, right coronary artery, and the left anterior descending artery-right ventricle fistula connection. (*E*) Cardiac magnetic resonance imaging showing a four-chamber view with the right atrium, right ventricle, left atrium, and left ventricle, confirming the absence of an right ventricle thrombus. (*F*) Follow-up coronary angiography demonstrating restored blood flow to both the left anterior descending artery and left circumflex artery (indicated by dashed arrows). LA, left atrium; LAD, left anterior descending artery; LCX, left circumflex artery; LV, left ventricle; RA, right atrium; RCA, right coronary artery; RV, right ventricle.

A 31-year-old woman with pulmonary atresia with an intact ventricular septum (PA/IVS) and a history of atriopulmonary Fontan conversion to total cavopulmonary connection at 27 years of age presented to the emergency department with acute chest pain and dyspnoea 3 h before admission. She had a known large sinusoidal communication between the right ventricle (RV) and left anterior descending artery (LAD). Her vitals and physical examination findings were unremarkable. She was receiving aspirin and warfarin; however, her prothrombin time–international normalized ratio was subtherapeutic at 1.3. Electrocardiography revealed ST-segment elevations in leads I and aVL (*Figure 1A*). The troponin T level was 0.779 ng/mL on admission. Coronary angiography (CAG) demonstrated a dilated LAD with to-and-fro flow, systolic flow into the aorta, and left circumflex artery (LCX) occlusion (*Figure 1B*, Supplementary material online, *Video S1*). Thrombectomy retrieved a red thrombus (*Figure 1C*), followed by dilation with a 1.5-mm balloon. Three-dimensional computed tomography revealed an LAD–RV fistula (*Figure 1D*), and cardiac magnetic resonance imaging confirmed no RV thrombus (*Figure 1E*). Follow-up CAG demonstrated restored distal LAD and LCX flow (*Figure 1F*). Thereafter, despite optimal medical therapy, the left ventricular ejection fraction declined from 50% to 29%, and New York Heart Association class III symptoms persisted. These findings suggested simultaneous occlusion of the LCX and distal LAD. The characteristic LAD flow pattern, sinusoidal communication, and dual coronary occlusion suggested retrograde embolization from the RV rather than antegrade embolization. Previous reports have described coronary artery occlusion via sinusoidal communication in post-Fontan patients with PA/IVS complicated by RV thrombus,^1^ and sinusoidal communication in PA/IVS has been associated with worse left ventricular function.^2^ Thus, this case highlights the importance of strict anticoagulation therapy in patients with PA/IVS with retrograde flow, even in the absence of prior thromboembolism or a detectable RV thrombus, given the potential for severe coronary events.

## Supplementary Material

ytaf208_Supplementary_Data

## Data Availability

The data underlying this article are available within the article.
